# Household water treatment and the nutritional status of primary-aged children in India: findings from the India human development survey

**DOI:** 10.1186/s12992-018-0356-7

**Published:** 2018-04-17

**Authors:** Wei Li, Echu Liu, Rhonda BeLue

**Affiliations:** 1 Department of Statistics, School of Data Science, Zhejiang University of Finance and Economics, Hangzhou, China; 2 Department of Health Management and Policy, College of Public Health and Social Justice, Saint Louis University, 3545 Lafayette Ave, Saint Louis, MO 63104 USA

**Keywords:** Household water treatment, Nutritional status, Children, India

## Abstract

**Background:**

Poor water quality, one of the leading causes of diarrhea, is an issue for most developing countries. Although the health burden of poor-quality water has been studied extensively, there is a paucity of research regarding the impact of household water treatment (HWT) on children’s nutritional status using data from large-scale surveys. In this research, we study the effect of HWT on the nutritional status of primary-aged children in India using a secondary data set consisting of 20,315 children between the ages of 6 and 14 (10,523 males and 9,792 females) in 12,839 households from the second wave of the India Human Development Survey (IHDS-II).

**Methods:**

The IHDS-II is a nationally representative, household-based, comprehensive, and face-to-face survey. Households were selected using stratified random sampling, and a team consisting of one male and one female interviewer visited each household between November 2011 and October 2012. A knowledgeable member, typically the male head of household, was interviewed about the socioeconomic condition of the household. An ever-married woman between the ages of 15 and 49, typically the wife of the male head of household, answered questions related to education and health. The height and weight of all eligible household members were measured by interviewers. Correlation between HWT and nutritional status was computed first, and the estimation of a generalized simultaneous equation model, in which a binary indicator of HWT and other covariates was included, was carried out afterward.

**Results:**

Bivariate analysis shows a negative association between the nutritional status of children and HWT. Additionally, findings from the generalized simultaneous equation model demonstrate that HWT increases the probability of producing normal-weighted primary-aged children by 1.7 %, while it decreases the probability of primary-aged children being thin by 2.5% and being severely thin by 1.7% in India.

**Conclusions:**

This study indicates that HWT has the potential to advance the nutritional status of primary school-aged children in India.

## Background

Water quality is always a critical public-health concern, especially in developing countries. As a developing country with the world’s fastest growing major economy [1] as well as the second largest population, poor water quality has been an ongoing problem [2, 3] in India because the country lacks water-treatment facilities that can handle the pollution caused by rapid industrialization and urbanization [4]; open defecation in many areas without sanitation infrastructure exacerbates the problem [5]. The negative effects of water supply are of increasing concern for people and policy makers with regard to human health at regional and national levels [6–10]. Therefore, water treatment is becoming particularly important in improving the quality of water for cooking or drinking and decreasing human-health risks. Considering the challenges and costs of managing the public water-supply infrastructure, household water treatment (HWT) has been regarded as an important and frontline procedure to achieve safe water supply in India [11].

Malnutrition among children continues to be a critical public-health issue in developing countries, and India is no exception. In fact, an estimated 39% of children between the ages of 0–59 months were stunted, and 29% were underweight, while 62.5% of the adolescent girls aged 10–18 were severely or moderately thin between 2013 and 2014 in India [12]. Despite some governmental policies and measures implemented to improve children’s nutritional outcomes, children’s nutritional status is still a serious and concerning public-health problem in India [13, 14].

As pointed out in the literature, drinking unclean water is a major contributing factor to diarrhea, an illness that claims numerous children’s lives in developing countries because contaminated water contains pathogenic bacteria, viruses, and parasites that can cause gastrointestinal diseases [15]. Moreover, diarrhea increases malabsorption, in turn increasing the likelihood of malnutrition. Therefore, it can be hypothesized that water quality in the household has a significant impact on children’s nutritional status in India.

The effect of water quality on children’s risk of diarrhea in India has been well documented [16–18]. However, studies on the impact of water on children’s nutritional status in India are relatively rare. One study found that the quality of piped water positively influences 627 children’s weight-for-age and weight-for-height in Madras, India, but the magnitude and significance of this effect vary across age groups [19]. Another study has demonstrated the benefits of access to piped water in the household on the nutritional status of primary-aged children in India [7]. However, these two studies have a common limitation: they rely on either quality of piped water [19] or access to piped water [7] as an indicator of the quality of domestic water supply, an approach we believe is problematic because, in contrast to the situation in developed countries, piped water is a questionable source of drinking or cooking water in India. For example, more than half of the pipes in rural areas in India deliver untreated water [20]. Therefore, the effect of water quality on children’s nutritional status in India has still not been clearly established.

To better understand the effect of water quality on children’s nutritional status in India, this study uses individual-level data from the India Human Development Survey (IHDS) to investigate whether HWT accomplished by boiling, filtering via a purchased filter, using an AquaGuard, or adding chemicals has significant effects on the nutritional status of primary-age children. Given the sanitation habits and infrastructure of water treatment in India, we believe that HWT is a more reasonable indicator of water quality in India than piped water.

## Methods

### Data

The IHDS is a nationally representative, multi-topic survey in India. Thus far, two IHDSs have been conducted: one in 2004–2005 and the other in 2011–2012. In each survey wave, a range of information about households and individuals was collected, including demographic characteristics, socioeconomic status, anthropometry, health, etc. This paper used data from the 2011–2012 IHDS (IHDS-II) because it was the most recent round and the only one to include precise information about several variables used in our statistical analysis.

The IHDS-II used face-to-face interviews, and households included in the IHDS-II were chosen using stratified random sampling. The interviewers asked a knowledgeable person, typically the male head of household, questions related to the socioeconomic status of the household (members), including questions related to income, employment, consumption expenditure, and social capital. An ever-married woman between the ages of 15 and 49 in each household was interviewed about health, education, family planning, marriage, and gender relations in the household and community. Adolescents between the ages of 15 and 18 were interviewed about their education, employment, marriage, life skills, future planning, friendships, and risky confidential behaviors. The IHDS-II interviewers also measured and recorded he weights and heights of all eligible household members during the interviews.

The IHDS-II surveyed 204,569 individuals and 42,152 households, but there were only 36,554 respondents between the ages of 6 and 14. Ultimately, only 20,315 from this sample were used in our study due to a lack of information on independent or dependent variables in some observations.

### Children’s nutritional status

In line with the World Health Organization’s (WHO) child-growth standards, children’s nutritional status in this study was assessed by BMI (body mass index)-for-age z-scores, which are defined by the number of standard deviations that a child’s BMI is above or below the median of BMI of the reference population provided by the WHO/National Center for Health Statistics (NCHS). In other words, a child’s BMI-for-age z-score was calculated using the following formula:

$$ \frac{Measured\ value\ of\  BMI- Median\ value\ of\  BMI\  of\ the\ reference\ group}{Standard\ deviation\ of\  BMI\  of\ the\ reference\ group} $$

After each individual child’s z-score was computed, his or her nutritional status is defined by one of the following integer values: 1 (obese if z-score > 2), 2 (overweight if 1< z-score ≤ 2), 3 (normal if −2 ≤ z-score ≤ 1), 4 (thin if −3 ≤ z-score < − 2), or 5 (severely thin if z-score < − 3).

### Household water treatment (HWT)

To measure if the water of a household in which a child resides is treated, we created a dichotomous variable (HWT) based on answers to this question from the IHDS-II: “During a normal week, do you ever treat or purify your drinking water by boiling the water OR by filtering the water with a purchased filter OR by using AquaGuard OR by adding chemicals?” Answers to this question are classified into four categories with adjective descriptions: 1 (never), 2 (rarely), 3 (usually), and 4 (always). HMT equals 1 if the answer is 3 (usually) or 4 (always) and 0 if the answer is 1 (never) or 2 (rarely).

### Incidence of diarrhea

It is impossible to achieve precise estimates of the magnitude and significance of the connection between HWT and children’s nutritional status without taking the effect of diarrhea into consideration in light of the relationship between water quality and nutritional status described in the background section. Hence, we created a binary indicator (diarrhea) based on answers to the following question from the IHDS-II: “Have you had diarrhea in the last 30 days?” Answers to this question are classified as 1 (yes) and 0 (no). Diarrhea equals 1 if the answer is yes and 0 if the answer is no.

### Controlled variables

Several variables were controlled for in our statistical analysis, including number of children in the household, number of meals in the household per day, hours of TV watching per day on an average day, gender (1 if male; 0 if female), whether the household income is below the poverty line (1 if yes; 0 if no), dummies for the highest education level of adults in the household (less than elementary school; elementary school; middle school), dummies for caste categories (Brahmin, Forward/General (except Brahmin), Other Backward Castes (OBC), Scheduled Castes (SC), and Scheduled Tribes (ST)), whether cooking is generally done outdoors (1 if yes; 0 if no), whether the household has a toilet (1 if yes; 0 if no), whether hands are washed after defecating (1 if yes; 0 if no), and 32 state dummies. Table 1 shows the descriptive statistics for all variables in our analysis except the 32 state dummies.

**Table 1:** Summary statistics of dependent and independent variables (*N* = 20,315)

Variables	Mean	Standard Deviation
Nutritional status (*n*_*i*_)	3.124	0.831
Having diarrhea in the last 30 days (*Diarrhea*_*i*_) (=1 if yes; =0 otherwise)	0.023	0.150
Household water treatment (*HWT*_*i*_) (=1 if yes; =0 otherwise)	0.178	0.382
Number of children in the household	2.838	1.498
Number of meals per day in the household	2.754	0.576
Hours of watching TV per day on an average day	2.079	1.309
Gender (=1 if male; =0 if female)	0.518	0.500
Household income is below poverty line (=1 if yes; =0 otherwise)	0.226	0.419
Highest education of adults in the household: less than elementary school (=1 if yes; =0 otherwise)	0.149	0.356
Highest education of adults in the household: elementary school (=1 if yes; =0 otherwise)	0.536	0.499
Highest education of adults in the household: middle school (=1 if yes; =0 otherwise)	0.149	0.356
Caste category: Brahmin (=1 if yes; =0 otherwise)	0.050	0.218
Caste category: Forward/General (except Brahmin) (=1 if yes; =0 otherwise)	0.209	0.407
Caste category: Other Backward Castes (OBC) (=1 if yes; =0 otherwise)	0.430	0.495
Caste category: Scheduled Castes (SC) (=1 if yes; =0 otherwise)	0.233	0.423
Caste category: Scheduled Tribes (ST) (=1 if yes; =0 otherwise)	0.070	0.254
Cooking in the household is generally done outdoors (=1 if yes; =0 otherwise)	0.201	0.401
Having a toilet at home (=1 if yes; =0 otherwise)	0.519	0.500
Washing hands after defecating (=1 if yes; =0 otherwise)	0.972	0.166

Note: the poverty line, which varies by state and urban/rural residence, is based on calculations of income needed to support minimal calorie consumption in 1970s and was adjusted by price indices since then

### Statistical analysis

**Fig. 1:**
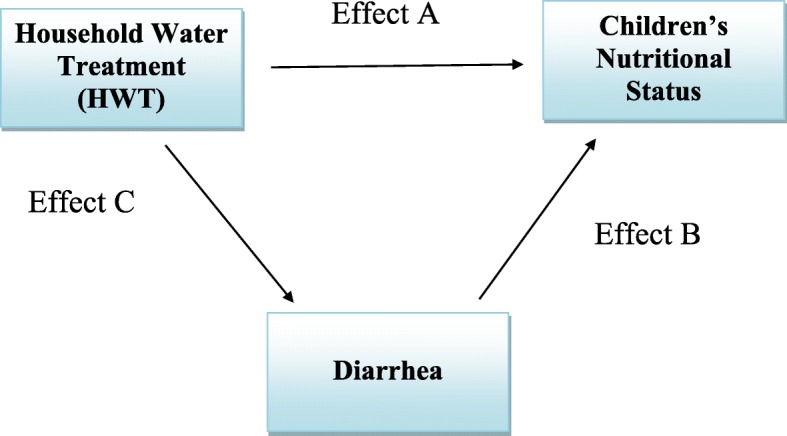
Connection between household water treatment (HWT) and children’s nutritional status

Our statistical analysis was carried out in two steps. First, the association between children’s nutritional status and HWT was examined to give us a rough idea of the empirical relationship between the two. Treating HWT as an indicator of water quality and given the relationship between water quality and nutritional status explained in the background section, the effect of HWT on children’s nutritional status can be decomposed into direct effect (effect A in Fig. 1) of HWT and HWT’s indirect effect via diarrhea (effect B × effect C in Fig. 1). In light of this decomposition and to assess the effects of HWT, we subsequently estimate a generalized simultaneous equation model, in which HWT, incidence of diarrhea, and all confounding factors described in the previous subsection were controlled, to assess the effects of HWT.

The generalized simultaneous equation model consists of two equations that define the relationship between HWT and diarrhea on one hand and diarrhea, HWT, and children’s nutritional status (*n*_*i*_) on the other. Mathematically, our generalized simultaneous equation model is specified as follows:

1 $$ {n}_i^{\ast }={HWT}_i\pi +{Diarrhea}_i\beta +{x}_i\gamma +{\varepsilon}_{i\kern0.75em } $$

2 $$ {Diarrhea}_i^{\ast }={HWT}_i\lambda +{z}_i\rho +{\epsilon}_{i\kern0.75em } $$

where ${n}_i^{\ast }$ is an underlying latent variable representing an individual child *i*’s propensity to be in a specific status of nutrition *n*_*i*_, which takes one of the following integer values: 1 (obese), 2 (overweight), 3 (normal), 4 (thin), and 5 (severely thin). ${Diarrhea}_i^{\ast }$ is an underlying latent variable representing an individual child *i*’s propensity to have diarrhea within the 30-day interval of the interview date. *Diarrhea*_*i*_ equals 1 if ${Diarrhea}_i^{\ast }>0$, and 0 otherwise. Variables *π*, *β*, *γ*, *λ*, and *ρ* are the regression parameters, and *ε*_*i*_ and *ϵ*_*i*_ are error terms that are assumed to follow a multivariate logistic distribution. *x*_*i*_ is the vector of independent variables that includes number of children in the household, number of meals in the household per day, hours of TV watching per day on an average day, whether the household income is below the poverty line, the highest education level of adults in the household, dummies for caste categories, and 32 state dummies. *z*_*i*_ is the vector of independent variables that includes whether the household income is below the poverty line, dummies for caste categories, whether cooking is generally done outdoors, whether the household has a toilet, and whether hands are washed after defecating. All parameters of interest, including *π*, *β*, *γ*, *λ* and *ρ*, are estimated jointly using maximum likelihood. Given that there are several binary regressors in the model, the marginal effect of HWT on nutritional status (*n*_*i*_), the marginal effect of diarrhea on nutritional status (*n*_*i*_), and the marginal effect of HWT on diarrhea were evaluated at a representative value of independent variables and used to estimate effect A, effect B, and effect C, respectively (see Fig. 1). Table 1 shows the summary statistics of all variables in our generalized simultaneous equation model.

## Results

### Bivariate analysis

Table 2 shows the distribution of our sample’s nutritional status by HWT. A higher percentage of children with access to treated drinking water are classified as “obese” and “overweight,” while a lower percentage of the same group of children are classified as “thin” and “severely thin” (compared to children who do not have access to treated water for drinking). Additionally, a lower percentage of children with access to treated water have a “normal” nutritional status (relative to those without such access). Overall, Table 2 shows there is a negative association between HWT and nutritional status among primary-aged children in India. However, although serving as a good starting point for our analysis, the results in Table 2 do not necessarily imply causality between these two variables, as no confounding factors have been taken into account.

**Table 2:** Nutritional status by household water treatment (HWT)

HWT	Nutritional status, in N (%)					
	Total	Obese	Overweight	Normal	Thin	Severely thin
1 (Yes)	3611	362(10.02)	366(10.14)	2242(62.09)	381(10.55)	260(7.20)
0 (No)	16,704	669(4.01)	910(5.45)	11,455(68.58)	2384(14.27)	1286(7.70)

### Generalized simultaneous equation model

The results of generalized simultaneous equation model are reported in Table 3. Tables 4, 5, and 6 report the estimates of effect A, effect B, and effect C, respectively, defined in Fig. 1, which is developed based on the relationship between water quality and nutritional status discussed in the background section. As Table 4 shows, HWT increases the probability of having a normal weight, being overweight, and being obese by 1.7%, 1.3%, and 1.1%, respectively. However, it decreases the probability of being thin by 2.5% and severely thin by 1.7%. Table 5 shows that having diarrhea decreases the probability of having a normal weight, being overweight, and being obese by 1.9%, 0.8%, and 0.6%, respectively. However, it increases the probability of being thin by 1.9% and severely thin by 1.4%, which is consistent with the fact that diarrhea increases malabsorption, which was mentioned in the background section. Table 6 shows that the impact of HWT on diarrhea is very small and statistically insignificant; hence, the indirect effect of HWT on nutritional status, which essentially mirrors what is reported in Tables 5 and 6, is almost negligible when calculating the total effect of HWT on nutritional status. As a result, the overall impact of HWT on children’s nutritional status (the sum of direct and indirect effects of HWT) reported in Table 7 is identical to those reported (effect A in Fig. 1) in Table 4. Based on the estimates reported in Table 7, children who consume water treated by boiling, filtering with a purchased filter, using AquaGuard, or adding chemicals in the household were less likely to have poor nutritional status than other children in India.

**Table 3:** Estimation results of generalized simultaneous equation model (*N* = 20,315)

Variables	Equation (1)	Equation (2)
Household water treatment (*HWT*_*i*_) (=1 if yes; =0 otherwise)	−0.262*** (0.045)	0.098 (0.125)
Having diarrhea in the last 30 days (*Diarrhea*_*i*_) (=1 if yes; =0 otherwise)	0.186* (0.096)	–
Number of children in the household	0.031*** (0.011)	–
Number of meals per day in the household	0.099*** (0.030)	–
Hours of watching TV per day on an average day	− 0.021* (0.012)	–
Gender (=1 if male; =0 if female)	0.176*** (0.030)	–
Household income is below poverty line (=1 if yes; =0 otherwise)	0.115*** (0.038)	− 0.230* (0.123)
Highest education of adults in the household: less than elementary school (=1 if yes; =0 otherwise)	0.079 (0.052)	–
Highest education of adults in the household: elementary school (=1 if yes; =0 otherwise)	− 0.078* (0.043)	–
Highest education of adults in the household: middle school (=1 if yes; =0 otherwise)	− 0.408*** (0.057)	–
Caste category: Brahmin (=1 if yes; =0 otherwise)	− 0.067 (0.180)	0.329 (0.536)
Caste category: Forward/General (except Brahmin) (=1 if yes; =0 otherwise)	− 0.135 (0.170)	− 0.355 (0.520)
Caste category: Other Backward Castes (OBC) (=1 if yes; =0 otherwise)	0.100 (0.168)	0.087 (0.511)
Caste category: Scheduled Castes (SC) (=1 if yes; =0 otherwise)	0.114 (0.169)	0.077 (0.516)
Caste category: Scheduled Tribes (ST) (=1 if yes; =0 otherwise)	0.158 (0.176)	−0.493 (0.558)
Cooking in the household is generally done outdoors (=1 if yes; =0 otherwise)	–	0.111 (0.117)
Having a toilet at home (=1 if yes; =0 otherwise)	–	−0.001 (0.101)
Washing hands after defecating (=1 if yes; =0 otherwise)	–	0.982** (0.452)
Log-likelihood = −23,117.747		

Notes: 1. Standard errors are in parentheses. 2. ***indicates *p* < 0.001; **p < 0.01; *p < 0.05. 3. Coefficient estimates on 32 state dummies are not reported

**Table 4:** Marginal effects of HWT on children’s nutritional status (effect A) (from eq. (1))

	Marginal effect
Prob (*n* = 1(obese))	0.011*** (0.002)
Prob (*n* = 2(overweight))	0.013*** (0.003)
Prob (*n* = 3(normal))	0.017*** (0.003)
Prob (*n* = 4(thin))	−0.025*** (0.004)
Prob (*n* = 5(severely thin))	−0.017*** (0.003)

Notes: 1. The representative sample includes those OBC male children who have two hours of watching television, three meals per day, habits of washing hands after defecating, and live in families with two children, toilets, doing outdoor cooking in general, highest adult educational attainment equal to elementary school (5–12 education years), and levels of household income below the poverty line in Uttar Pradesh. 2. *** indicates *p <* 0.01

**Table 5:** Marginal effects of Diarrhea on children’s nutritional status (effect B) (from eq. (1))

	Marginal effect
Prob (*n* = 1(obese))	−0.006** (0.003)
Prob (*n* = 2(overweight))	−0.008** (0.004)
Prob (*n* = 3(normal))	−0.019* (0.011)
Prob (*n* = 4(thin))	0.019* (0.010)
Prob (*n* = 5(severely thin))	0.014* (0.008)

Notes: 1. The representative sample includes those OBC male children who have two hours of watching television, three meals per day, habits of washing hands after defecating, and live in families with two children, toilets, doing outdoor cooking in general, highest adult educational attainment equal to elementary school (5–12 education years), and levels of household income below the poverty line in Uttar Pradesh. 2. ** indicates *p* < 0.05; * indicates *p* < 0.1

**Table 6:** Marginal effect of HWT on Diarrhea (effect C) (from eq. (2))

	Marginal effect
Prob (Diarrhea = 1)	0.001 (0.004)

Note: The representative sample includes those OBC male children who have two hours of watching television, three meals per day, habits of washing hands after defecating, and live in families with two children, toilets, do outdoor cooking in general, highest adult educational attainment equal to elementary school (5–12 education years), and levels of household income below the poverty line in Uttar Pradesh

**Table 7:** Overall impact of HWT on children’s nutritional status (effect A + (effect B x effect C))

	Marginal effect
Prob (*n* = 1(obese))	0.011
Prob (*n* = 2(overweight))	0.014
Prob (*n* = 3(normal))	0.017
Prob (*n* = 4(thin))	−0.025
Prob (*n* = 5(severely thin))	−0.017

## Discussion

### Main findings of this study

First, based on the average of HWT reported in Table 1, only approximately 17.8% of children in our sample are reported to have access to safe drinking water, which is low. Given that it has been reported that the use of HWT is related to household characteristics, such as education level and income level [21–24], and our sample consists of a high percentage of poor households with less-educated heads of household, this low percentage is not a surprise.

Second, based on the results from our generalized simultaneous equation model, it was found that HWT would decrease children’s chances of being “thin” and “severely thin,” while it would increase, although the effect is smaller, their chances of being “normally weighted,” “obese,” or “overweight” in India. Therefore, this study revealed that, in general, HWT increases children’s nutrition level in India. Our results make sense from a chemistry and epidemiology point of view. According to studies in these two fields, HWT is effective in terms of controlling volatile disinfection by-products, which are responsible for unsafe drinking water [25]. Additionally, treated drinking water by boiling in the household had a low risk of containing thermotolerant coliform compared to untreated water [26], and HWT systems constructed by inexpensive local materials consistently produce high-quality drinking water by removing waterborne bacteria [27]. Moreover, HWT filters are effective in the removal of turbidity and some contaminants [28].

As mentioned in the background section, literature of the relationship between water quality and nutritional status of children in India have a common limitation: they rely on either quality of piped water [19] or access to piped water [7] as a measure of water quality, but piped water is a questionable source of drinking or cooking water in India. Different from the literature, this study uses a more appropriate measure of water quality, which is HWT, to estimate the effect water quality on nutritional status of children in India, and hence a more precise estimate is anticipated to be generated.

### Limitations

This study has certain limitations. First, although we considered as many control variables as possible, some important factors, such as residential area, were not controlled for because they are presently restricted by the IHDS-II for public use. Furthermore, this research explored the effect of general HWT on children’s nutritional status and did not include modes of HWT in the analysis due to data limitations. An analysis that takes the modes of HWT into consideration will help us understand the difference in the efficiency of HWT with respect to improving children’s health in India once the data are available in the future.

## Conclusions

India still lags behind the United Nations Millennium Development Goals for childhood diarrhea and child malnutrition [29]. This study indicates that HWT decreases the probability of malnutrition for children in India. The findings in this study have important implications for the people and government of India. First, while HWT is effective, the Indian government should invest more heavily in improving access to potable water, such as scaling up well-structured, continuous piped water sources and improving access to HWT for families currently lacking reasonable-quality water supply. This is particularly important among poor households and poor regions/states that are disproportionately burdened by child malnutrition, morbidity, and mortality [30]. Furthermore, programs and policies are needed to communicate the importance of treating drinking water in order to prevent waterborne illness and associated diarrhea and malnutrition [29].

## Abbreviations

HWT: Household Water Treatment
IHDS: India Human Development Survey
NCHS: National Center for Health Statistics
WHO: World Health Organization
